# Sunscreen‐Inspired Nano‐Pesticide for UV‐Shielding and Laccase‐Responsive Delivery of Mancozeb With Enhanced Fungicidal Efficacy and Ecological Safety

**DOI:** 10.1002/advs.77197

**Published:** 2026-08-17

**Authors:** Jin Yan, Xin Ye, Linping He, Cheng Hong, Xu‐Huan Zhao, Gizachew Mulugeta Manahelohe, Xiao‐Tong Feng, Yu‐Cheng Gu, Qiang Bian, Min Wu, Ming‐Zhi Zhang

**Affiliations:** ^1^ Jiangsu Key Laboratory of Pesticide Science College of Sciences Nanjing Agricultural University Nanjing China; ^2^ The International Joint Institute of Tianjin University Fuzhou Tianjin University Tianjin China; ^3^ Department of Chemistry College of Natural & Computational Sciences University of Gondar Gondar Ethiopia; ^4^ National Pesticide Engineering Research Center (Tianjin) College of Chemistry Nankai University Tianjin China; ^5^ Syngenta Jealott's Hill International Research Centre Bracknell Berkshire UK

**Keywords:** laccase‐responsive, mancozeb, nano‐pesticide, photostability, sunscreen‐inspired nanocarrier

## Abstract

Fungicides are a crucial tool for protecting crops and ensuring consistent food supply. As a fundamental and widely‐used fungicide in global agriculture, mancozeb (MCB) suffers from rapid photodegradation, poor foliar adhesion, and substantial non‐target toxicity, limiting its efficacy and environmental compatibility. To address these issues, we developed a sunscreen‐inspired nano‐pesticide, MCB@URFSL, designed to enhance MCB's photostability, rainfastness, and targeted delivery. This system was fabricated via the co‐assembly of phthalate‐modified Pluronic F127 (URF) and lignosulfonate (SL), yielding monodisperse nanoparticles (∼180 nm) with integrated UV‐shielding, laccase‐responsive, and pathogen‐targeting properties. The strategy significantly improved MCB's photostability, retaining over 60% of antifungal activity after 5 days of UVA exposure. MCB@URFSL exhibited enhanced leaf adhesion and rainfastness on tomato leaves, along with laccase‐triggered release. In vitro and in vivo bioassays confirmed its superior protective and curative efficacy against fungal infections in cucumber seedlings. Importantly, the nanocarrier significantly reduced MCB's toxicity toward human keratinocytes, *Escherichia coli*, and zebrafish, with no acute mortality observed even at a concentration of 50 µg/mL. This work presents an intelligent nano‐pesticide strategy that synergistically enhances fungicidal efficacy and ecological safety, thereby establishing a promising platform for pioneering the next generation of agrochemicals.

## Graphical abstract

The sunscreen‐inspired nano‐pesticide, MCB@URFSL, provides UV‐shielding, enhanced rainfastness, and laccase‐responsive delivery for improved fungicidal efficacy and high ecological safety.

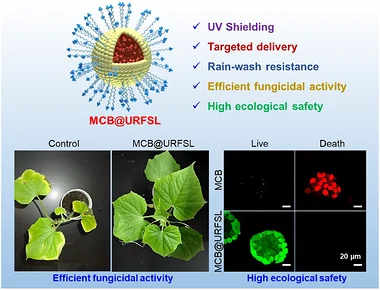

## 1 Introduction

Plant diseases pose a major threat to global food security, accounting for approximately 40% of annual crop losses worldwide [1, 2]. Conventional pesticide application remains the primary plant disease management approach [3, 4, 5]. As the leading dithiocarbamate fungicide, mancozeb (MCB) generates annual revenues exceeding $1 billion, dominating more than 70% of its market segment [6, 7]. MCB is a polymeric manganese zinc ethylene bis(dithiocarbamate) fungicide and is agriculturally indispensable due to its broad‐spectrum activity against over 70 fungal pathogens, including *Phytophthora*, *Alternaria*, and its multi‐site mode of action. These superiorities render MCB indispensable for controlling potato late blight, fruit scabs, and vegetable fungal diseases, and the Food and Agriculture Organization of the United Nations(FAO) classifies it as a medium resistance‐risk agent [8]. However, MCB suffers from rapid photodegradation (exceeding 90% within 48 h under UV irradiation), poor water solubility (0.006 g/L), and low foliar deposition (typically below 20%), which curtails its protective window from 7–10 days to a mere 3–5 days. In the open field, the sunlight‐driven degradation not only diminishes the fungicidal efficacy but also triggers an uncontrolled chemical cascade: the rapid cleavage of dithiocarbamate bonds causes a “burst” release of free manganese (Mn^2^^+^) and zinc (Zn^2^^+^) ions, generating high crop phytotoxicity and severe environmental footprints [9, 10]. These inefficiencies diminish its field efficacy and result in overusing, requiring 8 to 15 applications annually. Massive accumulation of MCB and their photolysis generating byproducts like ethylenethiourea (ETU) leads to significant ecotoxicity to aquatic organisms (fish LC_50_ = 1.2∼4.0 mg/L), soil microorganism (bacteria, fungi, and actinomycetes), and even human health [11]. Thus, developing innovative strategies to simultaneously improve fungicidal efficacy and mitigate risks of MCB is urgently needed.

Nanotechnology offers promising solutions for enhancing pesticide delivery while minimizing environmental destruction [12, 13, 14]. Nanomaterial‐encapsulated pesticides demonstrate improved bioavailability and enable stimulus‐responsive release [15, 16, 17], boosting utilization rates [18, 19]. Hence, the application of nanotechnology serves as a pivotal strategy for addressing the challenges related to MCB, facilitating the full exploitation of its fungicidal potential. Currently, nano‐formulations of MCB employ inorganic nanocarriers based on metal oxides such as tungsten oxide [20], silver oxide [21], and zinc oxide [22] as nanocarriers for bactericidal applications. Furthermore, among these inorganic metal nanocarriers, only zinc oxide has been demonstrated to alleviate its photodegradation [22]. Others merely enhance its bactericidal activity without fundamentally addressing the issues of photolysis and poor water solubility. Moreover, the introduction of heavy metals may potentially lead to environmental concerns [23]. Organic nanocarriers based on chitosan‐carrageenan nanocomposites [24] demonstrate enhanced antifungal performance and reduced ecotoxicity. However, susceptibility of MCB to photolysis has not been adequately addressed. Therefore, it is urgently needed to develop an organic nanocarrier that simultaneously addresses the issues of photodegradation, low solubility, and ecotoxicity related to MCB.

Pluronic F127 (F127) is a widely used organic nanocarrier for drug delivery, known for its high drug‐loading capacity [25], tunable properties [26], low toxicity [27], and cost‐effectiveness [28]. In parallel, benzoate‐based ultraviolet absorbers including octyl salicylate [29], meradimate [30], and octinoxate [31] were commonly employed in sunscreens owing to their intrinsic UV‐absorbing capability [32]. Furthermore, sodium lignosulfonate (SL), an anionic surfactant derived as a by‐product of the pulp industry [33], was chosen for its ability to significantly enhance aqueous solubility [34], reduce nanoparticle size [35], and improve colloidal stability [36]. As a derivative of lignin, a natural polymer, SL exhibits high biocompatibility [37] and can be degraded by laccase [38] enzymes released during fungal infection in plants [39]. Thus, we propose that integrating these three components—through chemical or physical assembly—into a novel hybrid material as a nano‐carrier for MCB may comprehensively address the existing challenges.

Herein, we synthesized UV‐resistant phthalate‐conjugated F127 (URF) and prepared MCB‐loaded URFSL nanoparticles via nanoprecipitation (Scheme 1). The resulting nanoparticles showed small size, high leaf deposition, controlled release, and excellent dispersibility. Upon fungal infection, laccase degrades both plant cell walls and the SL‐based nanostructure, triggering rapid MCB release for targeted action. The encapsulation system exhibited high biocompatibility and low non‐target toxicity. Comprehensive evaluations of dispersibility, UV stability, adhesion, antifungal performance, and biosafety were carried out. The results collectively demonstrate that this strategy is expected to significantly improve both the utilization efficiency and ecological safety of MCB.

**SCHEME 1:**
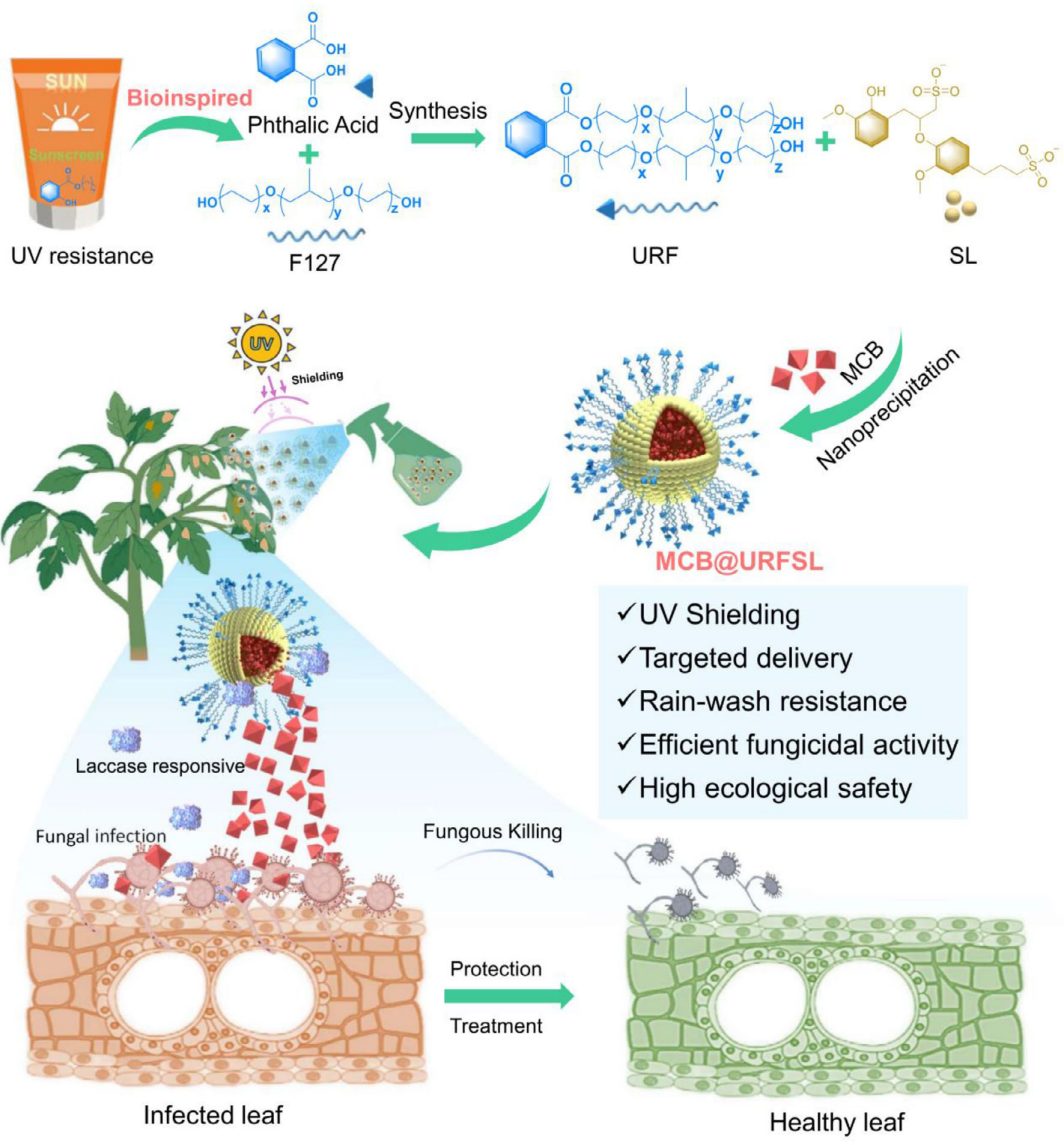
Schematic illustration of sunscreen‐inspired MCB@URFSL as a nano‐pesticide for combating fungal infection.

## 2 Results and Discussion

### 2.1 Preparation and Characterization of MCB@URFSL

The synthesis of URF with UV‐shielding capability was achieved via covalent interaction between F127 polymer with Phthalic acid. To verify the successful synthesis of URF, Fourier Transform Infrared (FT‐IR) spectroscopy and Hydrogen Nuclear Magnetic Resonance Spectroscopy (^1^H NMR) were carried out. As shown in FT‐IR spectrum (Figure 1B), URF displays a characteristic absorption between 1650 and 1770 cm^−1^ compared to F127, corresponding to the C═O stretching vibration. In addition, the ^1^H NMR spectrum (Figure S1) revealed additional aromatic proton signals in the region of 8–9 ppm for URF, which are absent in F127. Those results confirmed the successful conjugation of phthalic acid to F127.

**FIGURE 1:**
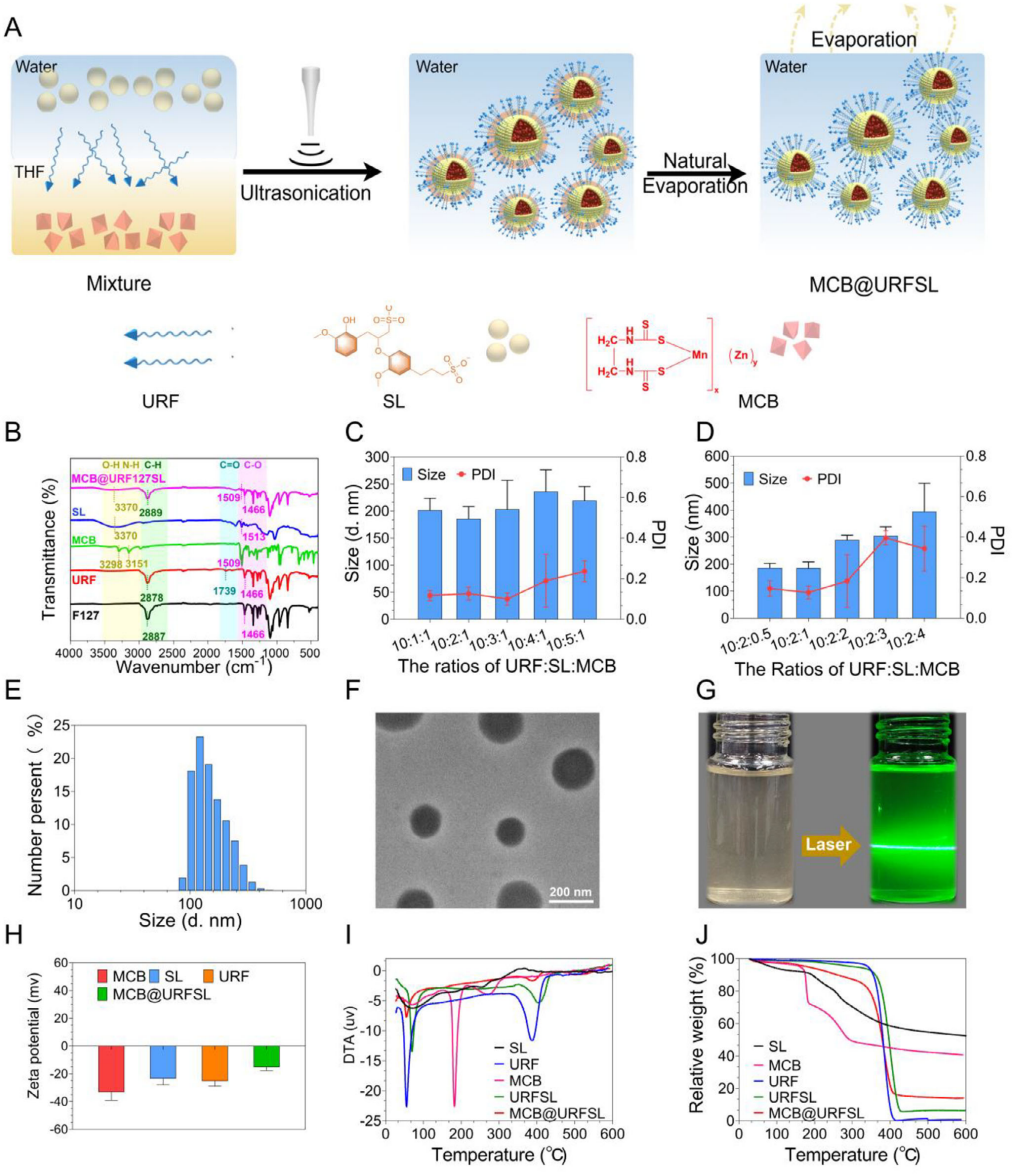
Preparation and characterization of MCB@URFSL. A) Schematic diagram of the MCB@URFSL preparation process. B) FTIR analysis of F127, URF, SL, MCB and MCB@URFSL. C,D) Size distribution of MCB@URFSL nanoparticles prepared with varying formulation ratios (n = 3 independent experiments, data are presented as mean values ± SD). E) Size distribution of MCB@URFSL. F) TEM images of MCB@URFSL. G) Photographs illustrating the Tyndall effect observed in MCB@URFSL. H) The zeta potential of MCB, SL, URFSL, and MCB@URFSL. I,J) Thermogravimetric and Differential Thermal Analysis of SL, URF, MCB, URF, MCB@URFSL.

The synthesis of MCB@URFSL with a distinct core‐shell structure was achieved via electrostatic interactions between MCB, SL, and URF using a nanoprecipitation method (Figure 1A). The functional groups on the MCB@URFSL surface were analyzed via FTIR spectroscopy, as shown in Figure 1B. The characteristic peaks of SL were observed in the spectrum of MCB@URFSL at 3370 cm^−1^, attributed to ─OH stretching vibrations, respectively. The characteristic peaks of MCB were observed in the spectrum of MCB@URFSL at 1509 cm^−1^, attributed to the ─C─N stretching vibration. The characteristic peaks of URF were observed in the spectrum of MCB@URFSL at 2889 cm^−1^, attributed to ─CH_2_─ stretching vibrations, respectively, and at 1466 cm^−1^, attributed to the ─C─H flexural vibration. Subsequently, to determine the optimal mass ratio of MCB, SL, and URF, the components were separately dissolved and then mixed at systematically varied ratios to prepare MCB@URFSL nano‐pesticides. As presented in Figure 1C,D, the nanoparticle size distribution of MCB@URFSL nano‐pesticides varied with varying mass ratios of MCB, SL, and URF. Previous studies have demonstrated that reducing pesticide particle size to approximately 200 nm can minimize foliar runoff, and it can more easily penetrate the cell walls of plant pathogenic fungi and enhance pesticide efficacy [40]. To identify the optimal carrier‐to‐cargo formulation, we systematically evaluated the trade‐offs between nanoparticle size, polydispersity index (PDI), encapsulation efficiency (EE), and loading content (LC) across a series of mass ratios of URF:SL: MCB (Figure 1C,D; Figure S2). At high active ingredient (AI) ratios (e.g., 10:2:3 or 10:2:4), the formulation suffered from a steep decrease in EE (below 50%) along with massive crystal precipitation, as the carrier concentration was insufficient to fully coat and stabilize the highly hydrophobic MCB core, leading to a rapid rise in nanoparticle size of MCB@URFSL (>400 nm). Conversely, relatively high polymeric carrier ratios (e.g., 10:2:0.5) achieved outstanding EE (∼85%) and extremely small sizes, but yielded an inefficiently low loading content, causing unnecessary carrier accumulation. By leveraging these parameters, a mass ratio of 10:2:1 (URF:SL:MCB) was identified as the optimized ratio. This specific formulation successfully harmonized a stable, uniform nano‐diameter (185.6 ± 20.35 nm), a narrow monodisperse distribution (PDI = 0.107 ± 0.032), a satisfactory encapsulation efficiency (68.5%), and a moderate loading capacity (5.3%), rendering it favorable for further applications. Therefore, the optimal mass ratio of URF, SL, and MCB was 10:2:1, and this formulation was selected for further experiments. At this ratio, the solution shows a distinct Tyndall effect (Figure 1G), and the particle size distribution (Figure 1E) also indicates a uniform MCB@URFSL dispersion. Transmission electron microscopy (TEM) images (Figure 1F) further corroborated the DLS results, showing consistent nanoparticle sizes and a regular, uniform spherical morphology. As indicated by the zeta potential analysis (Figure 1H), the value for MCB@URFSL was measured at approximately −15.19 mV. Although this moderate potential suggests that electrostatic repulsion alone is insufficient to maintain colloidal stability, the exceptional long‐term dispersion stability (no precipitation or aggregation over 60 days, as shown in Figure S3) is primarily attributable to steric stabilization. The hydrophilic polyethylene oxide (PEO) blocks of the URF block copolymer extend into the aqueous phase to construct a dense hydration barrier, creating a powerful steric hindrance that prevents nanoparticle coalescence. This steric effect is further augmented by electrosteric contributions from the outer lining of the lignosulfonate (SL) molecules, resulting in robust stability under agricultural preparation conditions. This repulsive force is sufficient to maintain a stable dispersed state over extended periods and effectively prevent sedimentation. The mass ratios of MCB to MCB@URFSL was 5.3% and achieving an encapsulation efficiency of 68.5%, which was measured by high‐performance liquid chromatography (HPLC) (Figure S4).

Thermogravimetric analysis (TGA) was employed to evaluate the thermal stability of MCB@URFSL nanoparticles. The sample was heated under a nitrogen atmosphere at a rate of 10°C per minute up to 600°C (Figure 1J). The mass loss observed at approximately 100°C was attributed to the evaporation of adsorbed and crystalline water. Differential Thermal Analysis (DTA) curves (Figure 1I) revealed the maximum weight loss temperatures: 184°C for MCB, 388°C for MCB@URFSL, 350°C for SL, and 400°C for URF, also confirming the successful combination of MCB with both URF and SL. Besides, MCB@URFSL nanoparticles exhibited high thermal stability, withstanding decomposition temperatures up to 380°C.

### 2.2 Stimulus Response Properties and Storage Stability of MCB@URFSL Nano‐Pesticide

Understanding the environmental response of MCB@URFSL nanoparticles is vital for optimizing their efficacy as nano‐pesticides. Leveraging the inherent sensitivity of SL to laccase, MCB@URFSL can specifically respond to laccase secreted by fungi and subsequently release MCB for rapid fungal eradication (Figure 2A). Therefore, we first calibrated MCB using HPLC (Figure S4), and subsequently investigated the in vitro release kinetics of MCB@URFSL in the presence or absence of laccase. As shown in Figure 2B, the presence of laccase significantly accelerated the release rate of MCB and resulted in a notably higher cumulative release compared to the control group without laccase. To decode the underlying physical transport mechanisms, the cumulative release curves were fitted using both the semi‐empirical Ritger‐Peppas and the classical Higuchi. Both curves showed high linear coherence with excellent goodness‐of‐fit. The fitting results reveal that the control group undergoes passive Fickian diffusion, while the experimental (+Laccase) group undergoes cooperative non‐Fickian anomalous transport, where the enzymatic degradation of the SL shell is synchronized with drug diffusion. The TEM images before and after laccase (Figure 2C,D) further demonstrated the laccase‐responsive release behavior of MCB@URFSL. Mechanistically, fungal laccase catalyzes the oxidative cleavage of ether bonds (predominantly β‐O‐4 and α‐O‐4 linkages) within the sodium lignosulfonate (SL) matrix, resulting in depolymerization of the protective shell and subsequent release of the encapsulated pesticide. Following release, the degraded low‐molecular‐weight lignin‐derived polyphenols serve as natural precursors to soil humus and undergo biotransformation and mineralization by indigenous soil microorganisms, preventing the long‐term accumulation of synthetic polymer residues [41]

**FIGURE 2:**
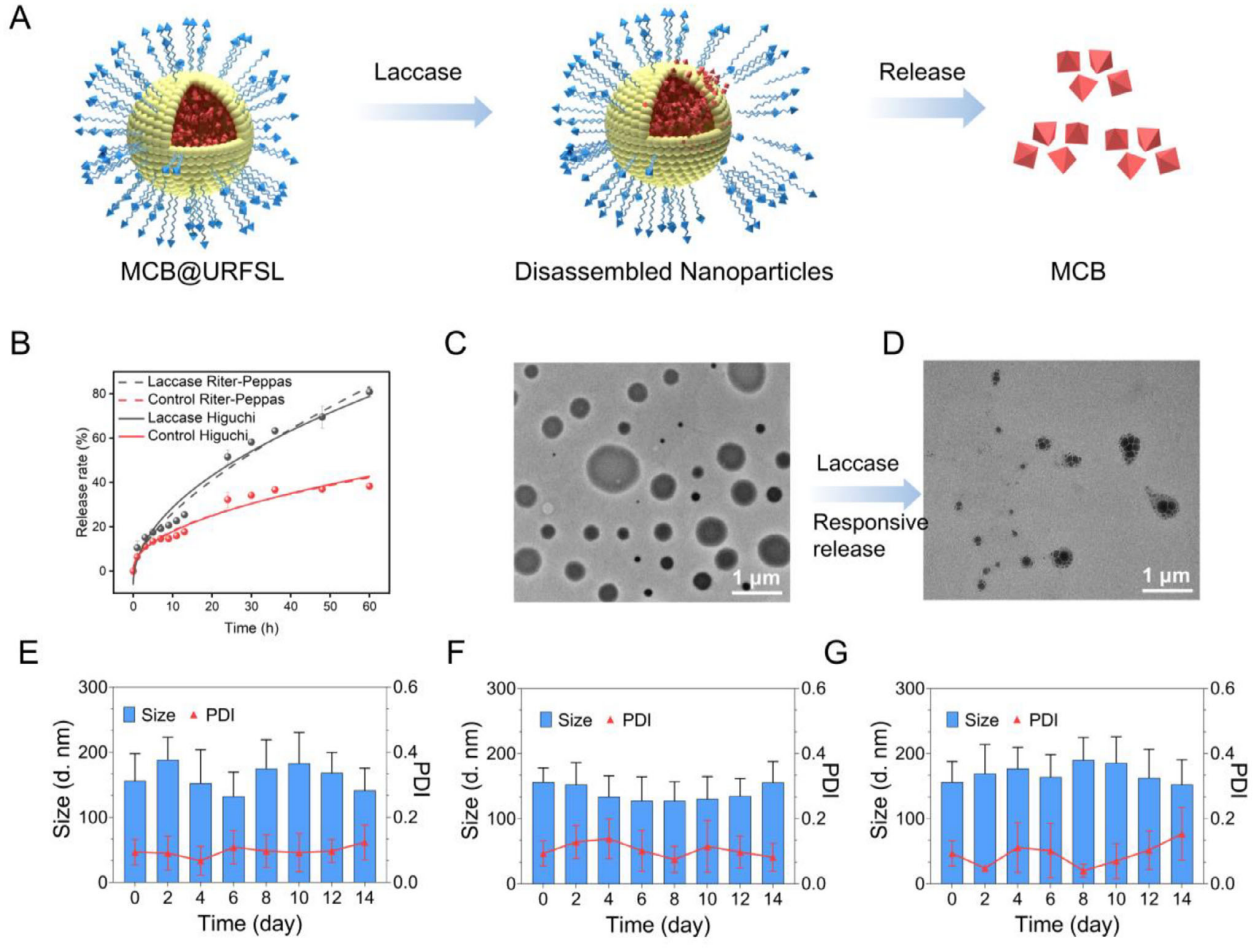
Stimulus responsive properties and storage stability of MCB@URFSL. A) Mechanism of laccase responsive release from MCB@URFSL. B) Kinetic modeling of the release behavior of MCB@URFSL under various environmental conditions; the results are expressed as mean ± SD (n = 3). C) TEM images of the MCB@URFSL release process. E–G) Changes in particle size of URF@MCB over 14 days at 25°C under UVA light, no light, and natural light; the results are expressed as mean ± SD (n = 3).

The particle size stability of nano‐pesticide emerges as a pivotal and expeditious criterion for screening and monitoring overall system integrity. It indicates whether the particles undergo significant degradation, aggregation, or size variation under storage conditions, thus providing insight into the potential preservation of bioactivity. The stability of MCB@URFSL was evaluated by monitoring the particle size and PDI following 14 days of storage at 25 °C under various conditions, including exposure to UVA light, no light, and natural light. As shown in Figure 2E–G, the particle size of MCB@URFSL retained within the range of 132.07 ± 37.28 nm to 189.91 ± 34.69 nm, with a PDI between 0.20 ± 0.09 and 0.009 ± 0.05, demonstrating good storage stability across all above ambient conditions.

### 2.3 Adhesion and Wettability of MCB@URFSL Nano‐Pesticides on Plant Surfaces

The adhesion and deposition of pesticide formulations on crop leaves significantly influence the efficacy of pesticide application [42]. Therefore, characterizing the wettability and adhesiveness of nano‐pesticides on plant surfaces is essential for improving their utilization efficiency [43]. As one of the commonly tested indicators in pesticide formulation studies, the Liquid Holding Capacity (LHC) is a key metric for evaluating the adhesion and retention of pesticide formulations on plant surfaces. It is critical for effective pest control, as it influences the persistence of active ingredients and their resistance to environmental factors such as rainfall. As shown in Figure 3A,B, the aqueous solution of URF exhibited a higher LHC compared to water, likely due to hydrogen bonding between the hydroxyl groups of URF and the leaf surface. MCB@URFSL demonstrated the highest LHC, which can be attributed to interactions between its abundant functional groups and fatty acids, alcohols, and aldehydes present in the leaf wax layer. Furthermore, the wettability and adhesion were evaluated by measuring the contact angles on fresh tomato leaves. The contact angles of water, MCB, URFSL, and MCB@URFSL were measured to be 90.9 ± 1.69°, 80.5 ± 3.2°, 67.6 ± 1.1°, and 64.9 ± 2.6°, respectively (Figure 3C,D). These results indicate that MCB@URFSL significantly enhances wettability and reduces droplet runoff compared to MCB.

**FIGURE 3:**
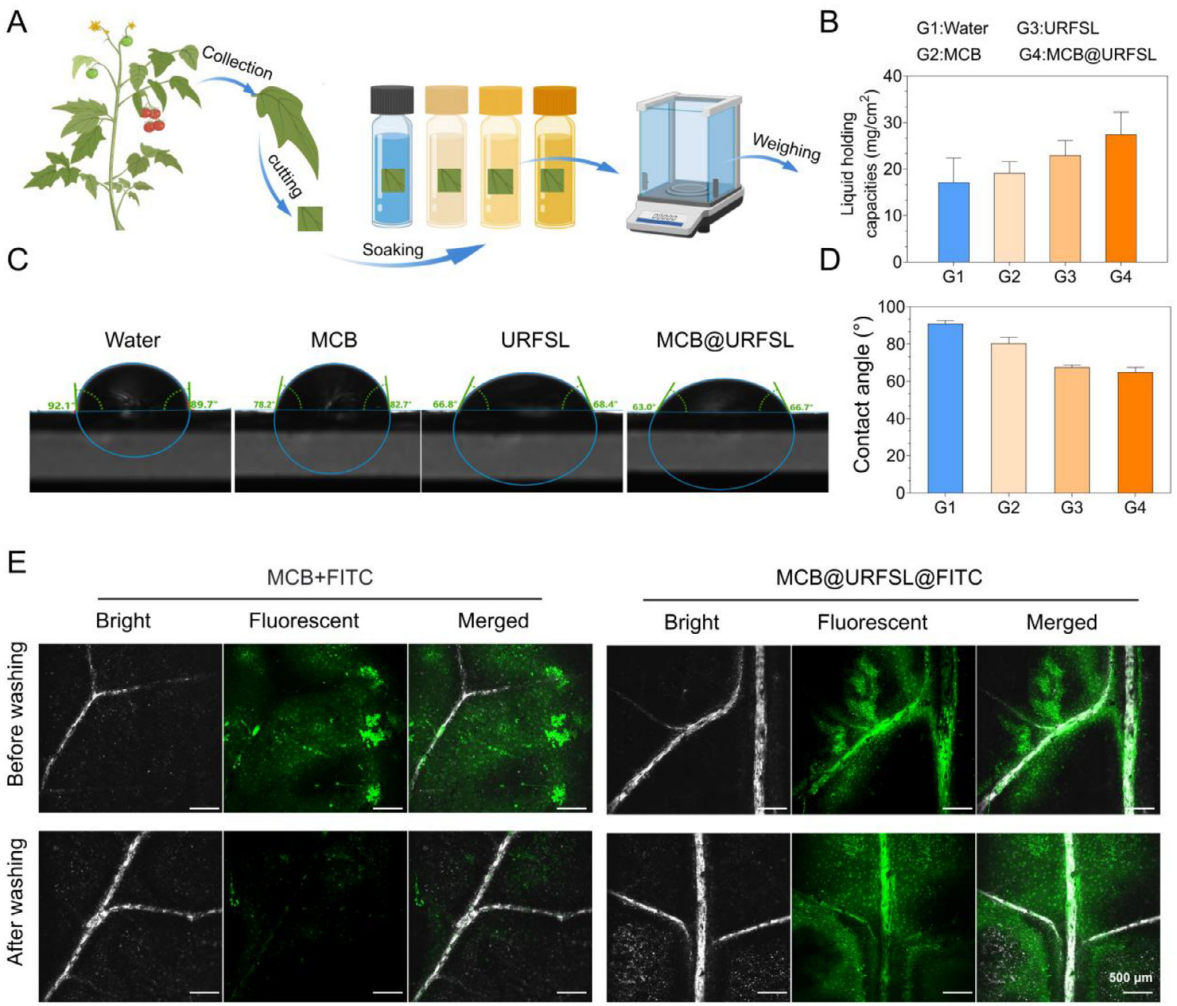
Wettability and Foliar Affinity of MCB@URFSL. A,B) Liquid holding capacities of tomato leaves after immersion in different solutions and corresponding experimental schematic diagram (n = 3 independent experiments, data are presented as mean ±SD). C,D) Contact angle images and data of 3 µL water, MCB, URFSL, and MCB@URFSL solutions measured at 30 s on tomato leaves using a contact angle meter (n = 3 independent experiments, data are presented as mean ± SD). E) Fluorescence images of MCB+FITC and MCB@URFSL@FITC solutions on leaves to simulate rain‐washing resistance (each experiment was independently repeated three times with similar results).

To evaluate rain wash resistance, FITC‐COOH was conjugated to URF using the same method, followed by dialysis and lyophilization to obtain URF@FITC. MCB@URFSL@FITC nanoparticles were then prepared using the previously described nanoparticle fabrication procedure. Fluorescence imaging of tomato leaves treated with either a mixture of FITC and MCB or MCB@URFSL@FITC was performed both before and after washing (Figure 3E). After rinsing, the fluorescence signal from the FITC and MCB mixture was very weak, indicating poor retention. In contrast, MCB@URFSL@FITC exhibited strong fluorescence, particularly along the leaf veins and epidermis, demonstrating excellent adhesion and rain wash resistance. The strong fluorescence of the veins is hard to be washed away by water. It is presumed that MCB@URFSL@FITC has entered the interior of the leaf.

### 2.4 UV Resistance of MCB@URFSL

MCB is susceptible to photolysis, leading to reduced bioavailability. What's worse, increasing the dosage of MCB may result in heightened toxicity to non‐target organisms, thereby significantly limiting its practical application. Therefore, improving its photostability is of significant importance. As shown in Figure 4A, MCB exhibits UV absorption in the range of 240 to 300 nm. However, as shown in Figure 4B, after being exposed to light for a period of time, the ultraviolet absorption at 273 nm will decrease significantly. To shield MCB from irradiation within this specific wavelength range, we conjugated a phthalate moiety to F127 to form URF. This modification enabled targeted UV absorption, thereby protecting the encapsulated MCB against photolysis. To evaluate the UV‐protective efficiency of URF, MCB and MCB@URFSL nanoparticles were respectively exposed UVA irradiation or under dark. Upon UVA irradiation, the UV absorption of free MCB at 273 nm started to decrease from the second day and had declined close to half of its initial value by the seventh day. In contrast, MCB@URFSL showed only a slight reduction on the first day, followed by a much slower decrease rate compared to MCB (Figure 4B,E). Even under dark conditions, free MCB underwent slight decomposition, whereas MCB@URFSL showed negligible degradation (Figure 4C,F). These results demonstrate that MCB@URFSL can effectively resist UV radiation‐induced decomposition of MCB.

**FIGURE 4:**
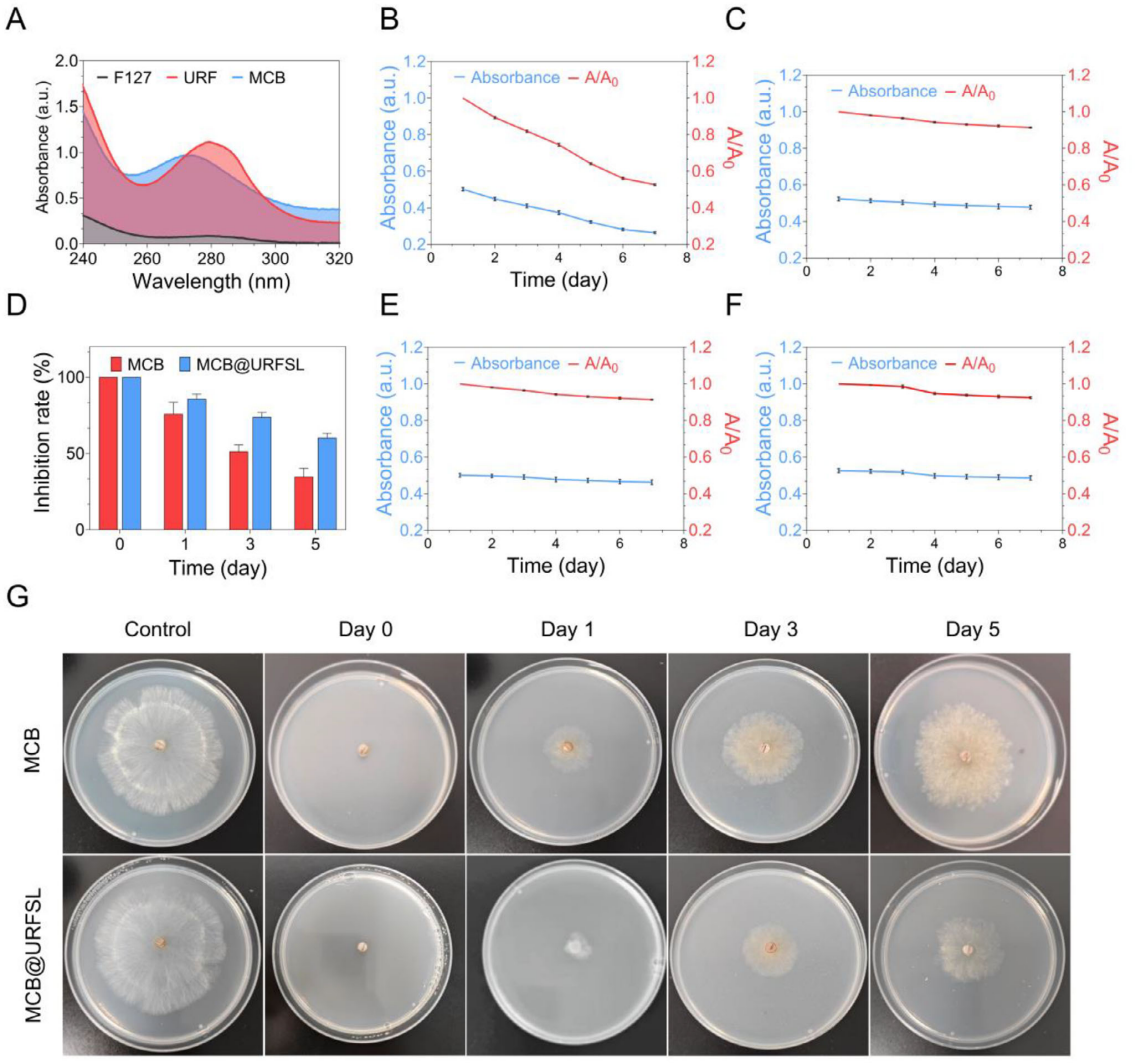
UV resistance and improved bioavailability of MCB@URFSL. A) UV–Vis absorption spectra of F127, URF, and MCB. B,E) The time‐dependent ultraviolet absorption variation of MCB (B) and MCB@URFSL (E) at 273 nm under UVA irradiation (n = 3, mean ± SD). C,F) The ultraviolet absorption of MCB(C) and MCB@URFSL(F) at 273 nm under dark changes over time (n = 3, mean ± SD). D,G) The antifungal activity of MCB and MCB@ URFSL following exposure to UVA light over varying durations (n = 3, mean ± SD).

To further confirm the enhanced bioavailability conferred by MCB@URFSL, antifungal activity assays of MCB and MCB@URFSL were conducted under UVA irradiation. As depicted in Figure 4D,G, the antifungal activity of free MCB began to decrease after one day of UV irradiation, while MCB@URFSL retained its antifungal efficacy (Figure 4D,G). After three days of irradiation, the antifungal activity of free MCB decreased by nearly 50%, whereas MCB@URFSL maintained approximately 75% antifungal efficacy. By the fifth day, free MCB retained less than 30% of its antifungal activity, whereas MCB@URFSL still exhibited nearly 60% inhibition against fungal growth. Collectively, these results demonstrate that URFSL can effectively mitigate the photodegradation of MCB, thereby promoting its bioavailability.

### 2.5 Plant Protection Conferred by Mcb@Urfsl Against Fungal Infection

Over a five‐day period, the in vitro antifungal efficiency of free MCB, URFSL, and MCB@URFSL against *Valsa mali* was evaluated at various concentrations, respectively. The results in Figure 5A,B demonstrated that the empty URFSL carriers exhibit negligible antifungal activity. MCB@URFSL exhibited approximately 100% antifungal activity when the concentration of MCB reached 25 µg/mL. However, free MCB showed less than 55% antifungal activity under the same conditions. These results indicated that MCB@URFSL exhibited significantly enhanced antifungal activity compared to free MCB, particularly at the low concentration, which was likely attributable to enhanced solubility and UV resistance of MCB following URFSL encapsulation.

**FIGURE 5:**
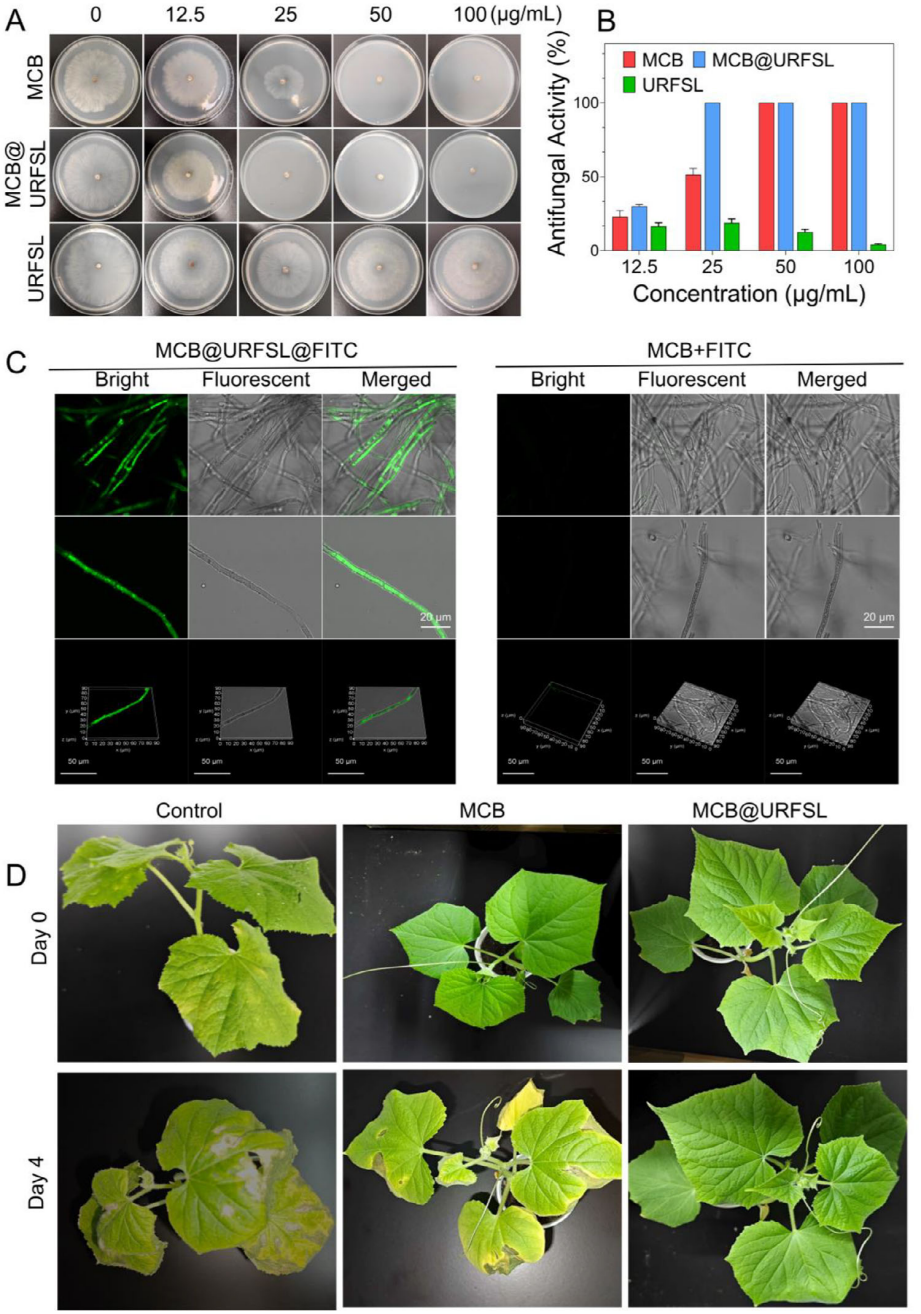
The application of MCB@ URFSL in plant protection against fungal infection. A) and B) In vitro antifungal activity to *Valsa mali* of MCB and MCB@ URFSL at various concentrations (n = 3, mean ± SD). C) Fluorescence images of MCB+FITC and MCB@URFSL@FITC solutions on infiltration fluorescence images of *Gibberella zeae*. D) Comparison of the protective effect between MCB and MCB@ URFSL against *Colletotrichum lagenarium* in pot experiments.

In addition, MCB@URFSL may facilitate a higher interfacial affinity and delivery efficiency of MCB to fungal cells, thereby enhancing its inhibition of fungal growth. To evaluate this leaf/pathogen boundary interaction, *Gibberella zeae* hyphae were incubated with either a physical mixture of free MCB/FITC or encapsulated MCB@URFSL@FITC (Figure 5C). The nanoparticles (MCB@URFSL@FITC) exhibited prominent green fluorescence along the hyphal structures, whereas free FITC exhibited negligible accumulation. This indicates that the amphiphilic block copolymer and lignosulfonate shell facilitate a close surface interaction (adsorptive affinity) and subsequent membrane internalization by the fungal mycelium. The results indicated that MCB@URFSL@FITC facilitated more effective internalization by hyphae, as evidenced by stronger green fluorescence from FITC, supporting the hypothesis that MCB@URFSL enhanced antifungal activity through improved fungal internalization.

The protective effect (PE, Figure 5D) and curative effect (CE, Figure S5) of plant against fungal infection mediated by MCB@URFSL were evaluated using indoor pot experiments. To assess the protective activity of MCB@URFSL against *Colletotrichum lagenarium* on cucumber seedlings, healthy seedlings were sprayed with 10 mL of a 50 µg/mL solution of either MCB or MCB@URFSL per three plants. After 24 h, allowing sufficient compound absorption, the plants were inoculated with a spore suspension of *C. lagenarium*. Four days post‐inoculation, the seedlings treated with MCB@URFSL retained unaffected. In contrast, those treated with free MCB showed partial leaf lesions but continued to grow, while the control group exhibited severe leaf damage and complete growth arrest. In the investigation of therapeutic efficiency, cucumber seedlings were first infected with *C. lagenarium* by inoculation with the spore suspension and then treated with 10 mL of a 50 µg/mL solution of MCB or MCB@URFSL per every three plants. Seven days after treatment, diseased leaves of seedlings treated with MCB@URFSL were effectively cured, and the plants resumed healthy growth. Seedlings treated with free MCB still showed visible lesions and delayed growth, whereas the control group displayed aggravated symptoms with severe disease progression. Collectively, MCB@URFSL markedly enhanced plant resistance to fungal infection and promoted the recovery of infected plants, demonstrating superior efficacy compared to free MCB.

### 2.6 Ecological Safety Evaluation of MCB@ URFSL

Ecological safety evaluation is indispensable in the development of new pesticides, providing critical insights into their environmental compatibility [44]. To assess the environmental safety of MCB@URFSL, its effects on human cell damage, bacterial toxicity, and acute/subacute toxicity in zebrafish were investigated. The Thiazolyl Blue (MTT) assay results shown in Figure 6B and Figure S6A demonstrate that MCB@URFSL exhibited negligible toxicity at concentrations below 6.25 µg/mL with the cell viability of *HaCat* cell lines exceeding 98%. Notably, even at a high concentration of 50 µg/mL, the cell viability retained above 71%. While Free MCB exhibited high toxicity toward *HaCat* cell lines, reducing cell viability to 55% at a concentration of 6.25 µg/mL and resulting in complete cell death at 50 µg/mL. As Live/dead staining assay results shown (Figure 6A), the cells treated with MCB exhibited complete cell death, as indicated by red fluorescence, whereas the cells treated with MCB@URFSL retained viable, showing green fluorescence, which corresponds to the results of MTT assay.

**FIGURE 6:**
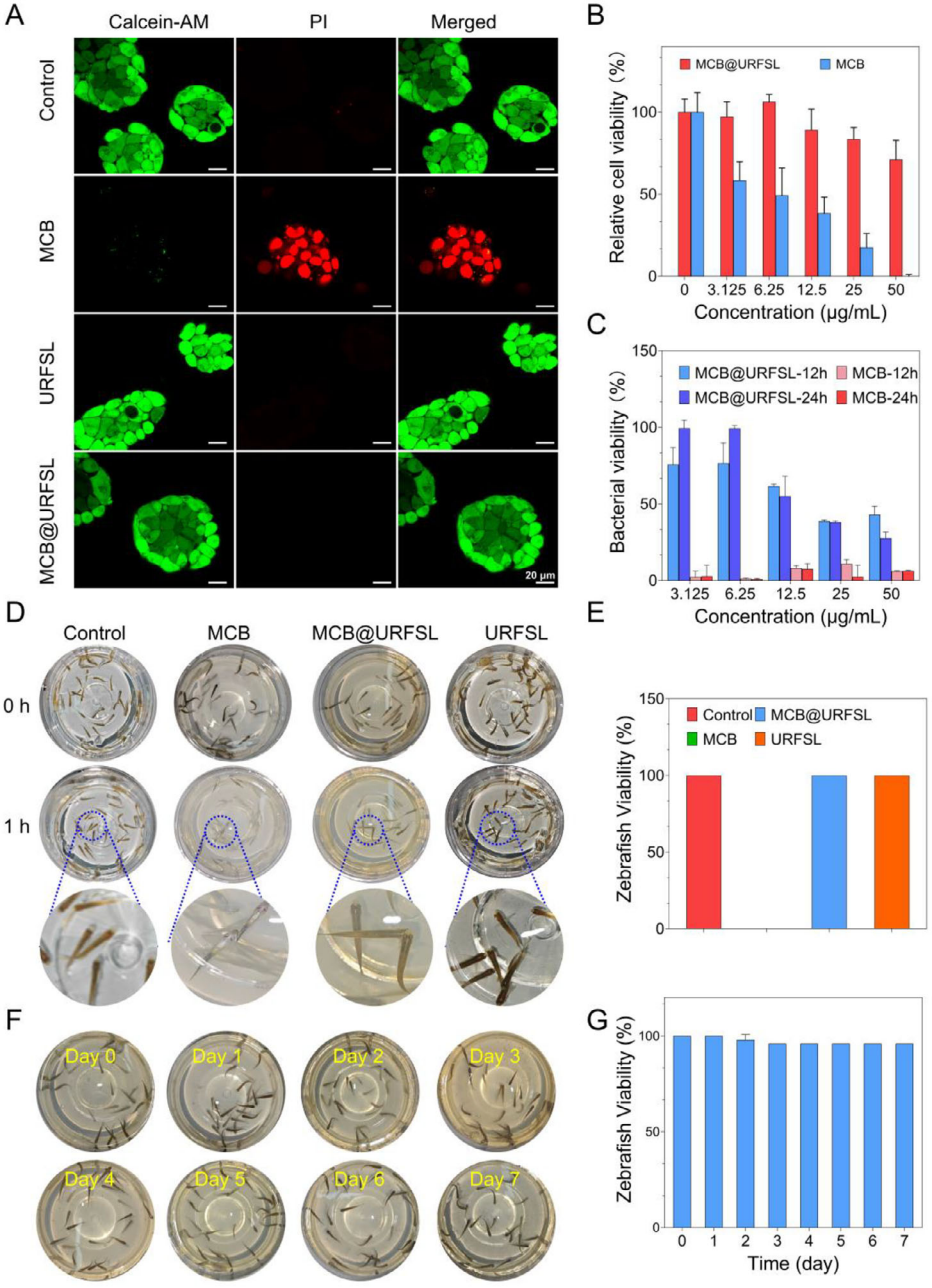
Assessment of ecological safety of MCB@URFSL. A) Images of living/dead cell staining for *HaCat* cells. Green indicates Calcein‐AM stained with living cells, red indicates PI stained with dead cell. B) MTT assay for *HaCat* cells (n = 3, mean ± SD). C) The bacterial viability of the *Escherichia coli* treated with MCB and MCB@ URFSL at various concentrations (n = 3, mean ± SD). D,E) The acute toxicity of MCB and MCB@ URFSL to zebrafish. F,G) The continuous toxicity of MCB@URFSL to zebrafish (n = 3, mean ± SD).

The toxicity of MCB@URFSL to *Escherichia coli* was evaluated by measuring OD_600_ at 12 and 24 h post‐treatment with various concentrations, serving as an indicator of its impact on bacterial microorganisms (Figure 6C; Figure S6B). The results demonstrated that encapsulation with URFSL effectively mitigated the toxicity of MCB to bacterial microorganisms. Notably, while free MCB showed high toxicity at low concentrations, MCB@URFSL exhibited almost no toxicity, thereby reducing its possible risk of disrupting the soil microbial environment caused by rain‐wash or leaching.

Furthermore, the acute and subacute toxicity of MCB@URFSL in zebrafish was evaluated. Exposure to 50 µg/mL of free MCB induced violent reactions within 10 min, including vomiting symptoms, and resulted in 100% mortality within 1 h, indicating its high toxicity. In contrast, no mortality was observed in groups treated with URFSL or MCB@URFSL at the same concentration (Figure 6D,E). Even after prolonged exposure time to MCB@URFSL for up to 7 days, no significant mortality was observed (Figure 6F,G). These results demonstrate that MCB@URFSL substantially reduces the toxicity of MCB to zebrafish, minimizing potential hazards to aquatic organisms. These results together confirm that encapsulation in the URFSL carrier significantly mitigates the acute toxicity of MCB toward representative dermal, aquatic, and non‐target microbial models. Although multi‐trophic assessments under field conditions remain essential to fully map its long‐term ecological footprint, this current ‘Safe‐by‐Design’ formulation offers a substantial improvement in farm‐level safety. (All applied concentrations of MCB@URFSL in the biological and safety assays correspond to the equivalent active ingredient concentration of mancozeb.)

Collectively, the MCB@URFSL nanoparticle system developed herein effectively addresses principal challenges associated with mancozeb application, including photolysis, poor adhesion, and localized crop phytotoxicity. Although further extensive multi‐trophic assays featuring key agricultural organisms (e.g., Eisenia fetida and beneficial insects) are warranted to comprehensively establish its long‐term soil compatibility, this bio‐based platform demonstrates significantly enhanced biosafety in representative models. This study provides a foundational framework for the design of next‐generation smart agrochemicals, harmonizing precision disease control with the overarching goals of green and sustainable agricultural systems. Furthermore, this rationally designed nano‐encapsulation strategy holds substantial promise for mitigating long‐term ecotoxicity in complex agricultural ecosystems. By constraining direct chemical exposure through physical nanocarrier encapsulation and minimizing spray drift via enhanced foliar adhesion, MCB@URFSL restricts off‐target pesticide runoff into soil and aquatic compartments. Furthermore, because active release is strictly gated by pathogen‐secreted laccase, the formulation avoids the uncontrolled initial burst release of toxic free Mn^2^^+^ and Zn^2^^+^ ions typical of technical mancozeb. This targeted behavior reduces the toxic burden on multi‐trophic soil microflora, earthworms, and aquatic communities, demonstrating the potential of smart nano‐pesticides to bridge laboratory safety findings to field‐scale ecological sustainability.

## 3 Conclusions

In conclusion, the MCB@URFSL nanoparticle system developed herein effectively addresses principal challenges associated with mancozeb application, including photolysis, poor adhesion, non‐target toxicity, and inefficient delivery. Through rational integration of UV‐protective and enzyme‐responsive constituents, this platform significantly enhances fungicidal performance while remaining environmentally friendly. This study provides a foundational framework for the design of next‐generation nano‐pesticides that combine targeted functionality with enhanced biocompatibility, harmonizing precision performance with overarching goals of agricultural sustainability.

## 4 Materials and Methods

### 4.1 Ethics Statement

All zebrafish experiments were conformed to the Zebrafish Information Network guidelines for the care and use of laboratory animals and were ethically approved by the Laboratory Animal Ethical Committee of Nanjing Agricultural University, NJAULLSC2025020.

### 4.2 Materials

Phthalic acid (98%), 2‐(7‐Azobenzotriazole)‐*N,N,N,N*‐tetramethylurea hexafluorophosphate (HATU) (98%), Tetrahydrofuran (AR 98%), ethyl alcohol (AR 98%), and 4‐dimethylaminopyridine (DMAP) (98%) were purchased from Adamas. Triethylamine (98%), Ultra‐dry dichloromethane (AR), Polyether F127 (98%), and sodium ligninsulfonate were purchased from Macklin (Shanghai, China). 5‐carboxyfluorescein (97%) was purchased from Energy Chemical.

#### 4.2.1 Microorganism and Cell Lines

The tested fungi were provided by the Laboratory of Plant Disease Control, Nanjing Agricultural University. *HaCat* call comes from Sunncell. *Escherichia coli* comes from ATCC. Calcein/PI Cell Viability/Cytotoxicity Assay Kit were purchased from Beyotime.

### 4.3 Method

#### 4.3.1 Synthesis of URFSL

In an ice‐water bath, phthalic acid was dissolved in a round‐bottom flask containing 5 mL of ultra‐dry DCM, along with 2 equivalents of HATU, 4 equivalents of triethylamine, and 0.2 equivalents of DMAP. After stirring for 30 min, 2 equivalents of F127 were added, and the reaction mixture was stirred overnight at room temperature. The reaction was terminated once TLC analysis indicated complete consumption of phthalic acid. The DCM was then removed under reduced pressure, and the resulting residue was dissolved in ultrapure water, then dialyzed using a 1000 Da dialysis bag, and lyophilized to afford URF.

#### 4.3.2 Preparation of MCB@URFSL

1 mg of MCB was weighed and dissolved in 500 µL of THF, and 2 mg of SL and URFSL were dissolved in 500 µL of THF. The two solutions were thoroughly mixed and then added at once into 10 mL of ultrapure water, undergoing ultrasonication with a probe‐type sonicator. After sonication for 5 min, THF was allowed to evaporate under light‐protected conditions with cooling. Ultrafiltration centrifuge tubes are used for ultrafiltration to remove free MCB, affording MCB@URFSL.

#### 4.3.3 Wettability and Foliar Affinity of Nano‐Pesticides

The adhesion of nano‐pesticides on leaves was evaluated by contact angle measurements. Fresh tomato leaves were rinsed with deionized water, air‐dried, and fixed onto glass slides. Droplets (3 µL) of different solutions were placed on the leaf surfaces, and contact angles were measured after 45 s using a contact angle meter (DSA100, KRUSS) [45].

Liquid holding capacity (LHC) was assessed by immersing 1 × 1 cm tomato leaf squares in various solutions (deionized water, 1 mg mL^−1^ MCB, URFSL, and MCB@URFSL) for 30 s, followed by weighing before and after immersion. Each measurement was repeated three times, and LHC was calculated using Equation (1), where M_0_ and M_1_ are the weights before and after soaking, and S is the leaf surface area [46].

(1) $$\text{LHC}=(M_{1}-M_{0})/S$$

where M_0_ and M_1_ represent the weights of the leaves before and after soaking, respectively, and *S* represents the surface area of the leaf sample.

To prepare FITC‐loaded nanoparticles, take 14.4 mg of FITC, add 15 mg of HATU, 2 mg of DMAP, and 300 µL of triethylamine, add 5 mL of DCM, activate for 30 min, then add 0.5 g of URF and react overnight. After the reaction, URFFITC was obtained by vacuum concentration and dialysis. The same method for preparing nanoparticles was used to obtain MCB@URFSL@FITC. Drops of FITC/MCB or MCB@URFSL@FITC (1 mL) were air‐dried naturally on the leaves, which were then sprayed with 5 mL of deionized water for 30 s. Changes before and after spraying were observed using confocal laser scanning microscopy (CLSM, Leica, STELLAIRS 5, Germany).

#### 4.3.4 In Vitro Antifungal Activity Assay

Antifungal activity was assessed via the mycelial growth rate method. MCB and MCB@URFSL were prepared as 100.00, 50.00, 25.00, 12.50, and 0 mg L^−1^ solutions and added to the culture medium. (MCB@URFSL solutions were prepared at equivalent mancozeb active ingredient.) *Valsa mali* disks were inoculated into culture media containing different concentrations of MCB and MCB@URFSL, using an equivalent volume of dimethyl sulfoxide (water or DMSO) as the blank control. Incubation was conducted at 25°C for 3–5 days. The fungal colony diameter was measured using the cross method, and the inhibition rate was calculated. DMSO was purchased from Aladdin Reagent Co., Ltd. (Shanghai, China) [47].

#### 4.3.5 Safety Evaluation

Toxicity Study of *HaCat* Cell. The effects of the MCB and MCB@URFSL on the activity of *HaCat* cells were detected using the Methylthiazol Tetrazolium (MTT) assay. The experimental the MCB and MCB@URFSL were prepared with a cell culture medium (DMEM: fetal bovine serum = 9:1) at different equivalent active mancozeb concentrations of 3.125, 6.25, 12.5, 25, and 50 µg/mL. The 20% DMSO positive control group and complete medium negative control group without pesticide were set up, respectively. The treated pesticide solutions were added to 96‐well plates (n = 3). The *HaCat* cells in the logarithmic growth phase were seeded in 96‐well plates at a concentration of 8 × 103/well and placed in a CO_2_ incubator (CLM‐170B‐8‐CN, ESCO). The *HaCat* cells were cultured at 37°C and 5% CO_2_ for 24 h and 48 h. After treatment with MTT (Beijing Soleibao Technology Co., Ltd., No.: M8180), the OD_570_ values were measured and recorded by a microplate reader (TECAN, Model: Infinite F50). The cell relative viability values were calculated at 48 h using the following Equation (2): [48]

(2) $$\mathrm{Relative}\;\mathrm{cell}\;\mathrm{viability}\;\left(\%\right)=\frac{OD_{i}}{\bar{OD_{c}}}\times 100\%$$

where OD_i_ is the OD_570_ value of the experimental group and $\bar{\;OD_{c}}$ is the average OD_570_ value of the negative control group.

#### 4.3.6 Microbial Toxicity Study

*E. coli* was diluted with LB broth medium. The mixtures were prepared with diluted bacterial solution and the MCB and MCB@URFSL at different EB equivalent active mancozeb concentrations of 3.125, 6.25, 12.5, 25, and 50 µg/mL. Mixtures of pesticide and Escherichia coli were added to 96‐well plates at different concentrations and incubated in a constant temperature incubator (MCO‐15AC, SANYO Electric Co., Ltd., Japan) at 37°C for 0, 12, and 24 h. The OD_600_ value was measured and recorded using a microplate reader (Synergy HTX, Burton Instruments USA, Inc.). The MCB and MCB@URFSL group was set as the control group (n = 3). The germ relative viability values were calculated at 12 and 24 h using the following Equation (3):

(3) $$\;\mathrm{Relative}\;\mathrm{germ}\;\mathrm{viability}\;\left(\%\right)=\frac{\bar{OD_{t}}-\bar{OD_{o}}}{\bar{OD_{it}}-\bar{OD_{io}}}\times 100\%$$

where $\bar{OD_{t}}-\bar{OD_{o}}$ is the OD_600_ value of the experimental group and $\bar{OD_{it}}-\bar{OD_{io}}$ is the average OD_600_ value of the negative control group

The biological safety of MCB@URFSL in aquatic organisms was determined using the zebrafish toxicity test. Zebrafish were acclimated under test conditions for >7days, with 12–16 h of light daily, regular removal of feces and leftover food, and a mortality rate < 5%. During acclimation, zebrafish were fed commercially available feed 1–2 times daily. Feeding was stopped 24 h before the test, and healthy and similarly‐sized individuals were selected for the experiment. A semistatic test method was employed, wherein MCB@URFSL were first prepared as a stock solution, and then the test solution was added to the fish tank, with water treatment as a blank control. Observations of toxicity symptoms and death counts were performed at 24 h and every 24 h later after the start of the experiment, with prompt removal of dead fish [49].

### 4.4 Statistical Analysis

Data processing, statistical analysis, and visualization are all conducted utilizing the Graphpad Prism 9.5 and Origin 2021. The data are presented as the mean ± standard deviation.

## Author Contributions

**Linping He**: methodology, software, data curation, investigation, validation. **Xu‐Huan Zhao**: methodology, software, data curation, investigation, validation. **Xin Ye**: software, data curation, methodology, investigation, validation, formal analysis. **Xiao‐Tong Feng**: methodology, software, data curation, resources, investigation, validation. **Ming‐Zhi Zhang**: conceptualization, methodology, software, data curation, investigation, validation, formal analysis, supervision, resources, project administration, visualization, funding acquisition, writing – original draft, writing – review and editing. **Yu‐Cheng Gu**: supervision, funding acquisition, conceptualization, methodology, project administration, resources. **Cheng Hong**: methodology, software, data curation, investigation, validation. **Min Wu**: conceptualization, methodology, software, data curation, investigation, validation, supervision, writing – original draft, writing – review and editing, visualization, formal analysis. **Gizachew Mulugeta Manahelohe**: writing – review and editing, conceptualization, validation, formal analysis, funding acquisition, visualization. **Jin Yan**: methodology, software, data curation, investigation, validation, formal analysis, writing – original draft, writing – review and editing, visualization, resources. **Qiang Bian**: methodology, data curation, resources, software, formal analysis, conceptualization, investigation, validation.

## Conflicts of Interest

The authors declare no conflicts of interest.

## Supporting information




**Supporting File**: advs77197‐sup‐0001‐SuppMat.docx.

## Data Availability

The data that support the findings of this study are available from the corresponding author upon reasonable request.
