# Supplementary material for: Sunscreen‐Inspired Nano‐Pesticide for UV‐Shielding and Laccase‐Responsive Delivery of Mancozeb With Enhanced Fungicidal Efficacy and Ecological Safety
Source: Adv Sci (Weinh). 2026 Aug 17:e77197. Online ahead of print. doi: 10.1002/advs.77197 (PMC13479584; doi:10.1002/advs.77197)
Supplement: Supplementary file 1 [file ADVS-9999-e77197-s001.docx]

**Supplementary Information**

**Sunscreen-inspired Nano-pesticide for UV-Shielding and Laccase-responsive Delivery of Mancozeb with Enhanced Fungicidal Efficacy and Ecological Safety**

Jin Yan^a,b^, Xin Ye^b^, Linping He^b^, Cheng Hong^b^, Xu-Huan Zhao^b^, Gizachew Mulugeta Manahelohe^a,c^, Xiao-Tong Feng^d^, Yu-Cheng Gu^e^, Qiang Bian^d^, Min Wu^b,^*, Ming-Zhi Zhang^a,^*

^a^ Jiangsu Key Laboratory of Pesticide Science, College of Sciences, Nanjing Agricultural University, Nanjing 210095, China

^b^ The International Joint Institute of Tianjin University Fuzhou, Tianjin University, Tianjin 300072, China

^c^ Department of Chemistry, College of Natural & Computational Sciences, University of Gondar, Gondar, 196, Ethiopia

^d^ National Pesticide Engineering Research Center (Tianjin), College of Chemistry, Nankai University, Tianjin 300071, China

^e^ Syngenta, Jealott’s Hill International Research Centre, Bracknell, Berkshire RG42 6EY, United Kingdom

Contents

[**Experimental Section**	1](#_Toc210170943)

[**Figures S1 to S6**	4](#_Toc210170944)

[**References**	6](#_Toc210170945)

# **Experimental Section**

*MCB@URFSL Nanoparticle Characterization*: Dynamic Light Scattering analyzer was performed using a NANOTRAC Wave II (Microtrac, UK) to determine the PDI，Zeta potentia and average particle size at a scattering angle of 90° and a temperature of 25 °C. Each value represented the mean of three measurements. The nanoparticles were measured in a stabilized state without dilution

*Thermal stability was assessed using thermogravimetric analysis* (TGA, Mettler Toledo SDTA851, Switzerland), with the temperature increasing from 40 to 600 °C at a rate of 10 °Cmin^−1^ under a nitrogen flow of 20 mL min^−1^.

*Encapsulation efficiency (EE%)***:** EE% was determined by centrifuging 4 mL of MCB@URFSL solution at 3500 rpm for 15 min. The supernatant was filtered through a 0.22 μm membrane, and the concentration of free MCB was quantified using HPLC (Waters E2695) with a XBridage-C^18^ column (50 × 4.6 mm, 3.5μm). The mobile phase was a 70:30 (v/v) mixture of water and methyl alcohol at a flow rate of 0.5 mL min^−1^, with detection at 270 nm.^[1]^ Encapsulation efficiency was calculated using a standard curve (Y = 495000*X + 11.50, R^2^=0.9990, 0.01–0.1 mg mL^−1^,

Equation (1).

EE = (**m** _total MCB_ −**m** _free MCB_)/**m** _total MCB_	(1)

where **m** _total MCB_ is the initial mass of all MCB in the nano-formulation before dialysis, and **m** _free MCB_ is the mass of unencapsulated MCB in the dialysate after dialysis.

*Storage stability of MCB@URFSL***.** To assess the storage stability of the nano-pesticides at room temperature, samples were maintained at 25°C for a duration of 14 days. At specific time points in time every two days, 200 μL of the sample was withdrawn for analysis. The mean particle size of the nanoparticles and the change in polydispersity index (PDI) were utilized to evaluate the stability of the formulations.^[2]^

*In Vitro Controlled Release*: MCB release from MCB@URFSL nanocarriers was assessed via dynamic dialysis. 5 mL of MCB@URFSL and adding the appropriate amount of laccase suspension was transferred into a dialysis bag (MWCO = 1000 Da). The bag was then immersed in 80 mL of Water and placed in a shaking incubator set to 120 rpm at a steady 25 °C. Periodically, 1 mL of the release medium was sampled to measure MCB concentration via HPLC (Waters E2695) with a XBridage-C18 column (50 × 4.6 mm, 3.5μm), with an equal volume of fresh medium added to maintain consistent volume. The mobile phase, a mixture of water and methyl alcohol (20:80 v/v), was run at a flow rate of 0.5mL min−1. Analysis was conducted at a wavelength of 270 nm.^[3]^ Standard procedures for release profile measurements are detailed in the Supporting Information. The cumulative release percentage of MCB was calculated using Equation 2:

**Cumulative release (%) =**$\sum_{\boldsymbol{t=0}}^{\boldsymbol{t}} \frac{\boldsymbol{M}_{\boldsymbol{t}}}{\boldsymbol{M}_{\boldsymbol{o}}}$***×100%***	(2)

where Mt is the amount of MCB released to each sampling time point, *t* is the time of the release of MCB and *M_0_* is the initial weight of the MCB-loaded in the MCB@URFSL nanocarriers.

*Control efficiency assay in pot experiment***:** Control efficacy for the M. grisea was conducted in vivo using pot experiments. Cucumber seedlings were selected as the host model plant. Stock suspensions of MCB and MCB@URFSL were diluted to achieve a final MCB concentration of 50 μgmL^−1^. Subsequently, 5 mL of each solution was sprayed onto cucumber seedlings. *C. lagenarium* spore suspensions (2 × 105 mL^−1^) containing 0.01% Tween-20 were sprayed onto cucumber seedlings at 0 (≈1 h), 7days following the sample treatments. Afterwards, the plants were maintained in an artificial climate chamber at 28 °C with a 12-hour light/dark cycle and a relative humidity of over 95. Control efficiency was calculated on the fifth day to record the pathogen infection status of cucumber seedlings.

*The mycelium is infected by drugs:* Hyphae of *Gibberella zeae* were treated with either a mixture of MCB and FITC or with MCB@URFFITCSL nanoparticles, followed by fluorescence imaging after a certain incubation period

*Anti-ultraviolet effect*: On the one hand to evaluate the UV-protective effect of URF, solutions of free MCB in water and MCB@URFSL nanoparticles were exposed to both UVA irradiation and dark conditions. Samples were taken at regular intervals to measure UV absorption and assess in vitro antifungal activity after light exposure. On the other hand, take the MCB and MCB@URFSL after a certain period of UVA light exposure above to determine the in vitro antibacterial activity.

*Live and death staining*: For cell apoptosis testing, log-phase cells were collected and seeded into 6-well plates at a density of 40000 cells/well. Cells were cultured at 37 °C under 5% CO_2_ until adherence. Various treatment solutions were added, and cells were cultured for 24 h. After collection, the cell precipitates were resuspended in prechilled phosphate-buffered saline and transferred to 1.5 mL Eppendorf tubes. Cells were stained using the PI/ Calcein-AM apoptosis detection kit for 15 min, then observe it with a confocal microscop (CLSM, Leica, STELLAIRS 5, Germany).^[4]^

# **Figures S1 to S6**

**Figure S1.** ^1^H NMR of F127(A, B) and URF (C, D) (400 MHz, DMSO-d_6_).

**Figure S2**. Loading content and encapsulation efficiency of MCB@URFSL nanoparticles prepared with varying formulation ratios (n = 3 independent experiments, data are presented as mean values ± SD)

**Figure S3.** Changes in particle size of URF@MCB over 60 days at 25 °C under no lightthe results are expressed as mean ± SD (n = 3)

**Figure S4.** MCB standard curve (A) and HPLC separation chromatogram (B).

**Figure S5.** Comparation of the curative effect between MCB and MCB@ URFSL against *Colletotrichum lagenarium* in pot experiments.

**Figure S6.** A) MTT assay for HaCat cells of URFSL. B). The bacterial viability of the *Escherichia coli* treatment at URF127SL at different concentrations.

# **References**

1	N. Cerioli, W. Bououdina, A. Mereu, et al.,"Reprogramming the melanoma and immunosuppressive myeloid cells with esomeprazole-loaded PLGA nanoparticles" *iScience*, *28* (2025): 111559, https://doi.org/10.1016/j.isci.2024.111559.

2	Y. Ma, M. Yu, Z. Sun, et al.,"Biomass-based, dual enzyme-responsive nanopesticides: eco-friendly and efficient control of pine wood nematode disease" *ACS nano*, *18* (2024): 13781, https://doi.org/10.1021/acsnano.4c02031.

3	J. Zhang, B. Lou, X. Qin, et al.,"Using amphiphilic polymer micelles as the templates of antisolvent crystallization to produce drug nanocrystals" *ACS Omega*, *7* (2022): 21000, https://doi.org/10.1021/acsomega.2c01792.

4	F. Nakagawa, H. Kawashima, T. Morita, et al.,"Water-soluble closo-docecaborate-containing pteroyl derivatives targeting folate receptor-positive tumors for boron neutron capture therapy" *Cells*, *9* (2020): 1615, https://doi.org/10.3390/cells9071615.
